# Disciplined Improvisation: Characteristics of Inquiry in Mindfulness-Based Teaching

**DOI:** 10.1007/s12671-014-0361-8

**Published:** 2014-11-29

**Authors:** Rebecca S. Crane, Steven Stanley, Michael Rooney, Trish Bartley, Lucinda Cooper, Jody Mardula

**Affiliations:** 1 Centre for Mindfulness Research and Practice, School of Psychology, Bangor University, Bangor, UK; 2 School of Social Sciences, Cardiff University, Cardiff, UK; 3 City and Hackney Primary Care Psychology Service, Homerton University Hospital Trust, London, UK

**Keywords:** Conversation analysis, Mindfulness-based stress reduction, Mindfulness-based cognitive therapy, Inquiry, Pedagogy, Integrity

## Abstract

Evidence for the effectiveness of mindfulness-based stress reduction (MBSR) and mindfulness-based cognitive therapy (MBCT) is rapidly growing as interest in this field expands. By contrast, there are few empirical analyses of the pedagogy of MBSR and MBCT. Development of the evidence base concerning the teaching of MBCT or MBSR would support the integrity of the approach in the context of rapid expansion. This paper describes an applied conversation analysis (CA) of the characteristics of inquiry in the MBSR and MBCT teaching process. Audio-recordings of three 8-week MBCT and MBSR classes, with 24, 12, and 6 participants, were transcribed and systematically examined. The study focused on the teacher-led interactive inquiry which takes place in each session after a guided meditation practice. The study describes and analyzes three practices within the inquiry process that can be identified in sequences of talk: turn-taking talk involving questions and reformulations; the development of participant skills in a particular way of describing experience; and talk that constructs intersubjective connection and affiliation within the group. CA enables fine-grained analysis of the interactional work of mindfulness-based inquiry. Inquiry is a process of disciplined improvisation which is both highly specific to the conditions of the moment it took place in and uses repeated and recognizable patterns of interaction.

## Introduction

There is an extensive empirical evidence base for the effectiveness of mindfulness-based stress reduction (MBSR) and mindfulness-based cognitive therapy (MBCT) (hereon abbreviated to MB) approaches as an intervention in clinical settings, education, and the workplace (e.g., Fjorback et al. 2011; Piet and Hougaard 2011). However, there is a dearth of studies concerning the pedagogical processes involved in teaching MB courses. Leaders in the field have expressed concerns about the potential for a dilution of integrity of the approach, in part because of lack of in-depth understanding about MB teaching practice (Williams and Kabat-Zinn 2011). There is a descriptive and theoretical literature on the pedagogy of MBSR and MBCT (Crane 2009; Kabat-Zinn 2013; McCown et al. 2010; Santorelli 2000; Segal et al. 2012). This practitioner literature would be strengthened by empirical studies of the MB teaching process. Gaining an insight into key aspects of the pedagogy would play an important part in understanding how the approach achieves its affects and how teacher training can effectively support the development of competence.

The only study to date which takes the teacher as the research object (van Aalderen et al. 2012) is a qualitative analysis of the role of the teacher in MBCT, involving interviews with course participants and teachers, a focus group of teachers, and an observational analysis of an MBCT course. Their findings offer support to the practitioner view that the teacher’s embodiment of mindfulness is a central way through which participant learning is facilitated.

Crane et al. (2012) developed a MB teaching competence framework and evaluated its psychometric properties (Crane et al. 2013). This work demonstrated that a group of experienced MB teachers can agree on a structured and consistent rubric for assessing MB teaching against criteria and that the tool can be used reliably by experienced MB trainers who are trained in its use.

There is also a small but growing body of research examining mediators of change in MB courses. For example, the Kuyken et al. (2010a) research established that cultivation of both self-compassion and mindfulness plays an important role in protecting participants from future depression. This suggests that pedagogical processes that support the development of self-compassion are likely to be important in supporting positive participant outcome and are therefore likely to be important areas of teacher competence. Trials are increasingly including analysis of mediator variables in their design (Huijbers et al. 2012; Kuyken et al. 2010b; Williams et al. 2010), so it is likely that studies of mechanisms of action will increase over the next few years and that this will feed the knowledge base on MB teaching.

In order to maximize outcomes and ensure fidelity to appropriate teaching standards, it is important to build on this initial knowledge base and develop systematic investigations of the pedagogy of the teaching process by directly studying it. There are, however, some intriguing tensions in examining the practice of MB teaching. The outcome evidence base is located within the scientific/medical paradigm, and some writers on mindfulness teaching argue that many of its dimensions lie outside that paradigm (McCown et al. 2010). Even if we accept that MB teaching is amenable to empirical examination, we still face methodological challenges. Many of these challenges would be familiar to researchers examining the process aspects of psychotherapy and education, and some are unique to a mindfulness context.

A number of methodologies can be used to analyze the key features of the conversational practice of inquiry in MBCT and MBSR. Applied conversation analysis (CA; Hutchby and Wooffitt 2009) is a particularly suitable method because it provides a naturalistic, observational investigation of the process. Antaki (2011) offered the following working definition of CA: “the close examination of language in interaction. It answers the concrete questions: how do you and I bring off the business we transact with each other?” (p. 2). Conversation analysts study social life through analysis of social interaction, specifically, turn design and interactional organization are investigated to understand how meaning, social action, and context are created moment-by-moment. CA imposes a discipline on the researcher to interpret how participants display their understandings of what is happening within interaction. This contrasts with a typical MB practitioner perspective of focusing on the intention that might have been behind a teacher’s utterance. CA allows a close examination of what speakers are observably and hearably doing with their talk through an investigation of recurrent patterns or practices of interaction.

The study focused on the practice of inquiry for two reasons. First, it is the aspect of MB teaching practice which trainees frequently identify as being the most challenging to develop skills in. The results therefore have the potential to inform teacher training developments. Secondly, we were interested in piloting the CA method and therefore chose to focus the research on the interactional aspects of MB teaching.

There is a well-developed practitioner literature which describes the process and pedagogical principles underpinning inquiry in MBCT and MBSR (Crane 2009; Felder et al. 2012; McCown et al. 2010; Santorelli 2000; Segal et al. 2012). An inquiry sequence is described in this literature as occurring in the following generic way.

Following the meditation practice, the teacher begins a conversation by asking participants what they noticed during the practice. They do this to encourage reflection and exploration on their experience; work together through dialogue about these observations to find out what is being discovered; and link these observations and discoveries to the learning themes of the program. Inquiry aims to reveal and bring into conscious awareness automated and unrecognized habits and patterns of thinking and feeling and to make known some of the properties of thoughts and feelings. The manner of attending to the experience, the teacher, and the relational process are all thus aiming to offer an embodiment of the attitudinal qualities of mindfulness. It is suggested that this supports participants to internalize a mindful way of relating to experience which includes increased levels of self-compassion, reduced levels of cognitive reactivity, and the development of a capacity to allow, rather than problem solve, whatever experience is present in the moment.

The study aimed to discover how MB inquiry sequences are actually conducted in practice by skilled MB teachers in collaboration with their participants. We aimed to investigate the interactional practices employed by experienced MB teachers in order to investigate: How do MB teachers lead inquiry? How are sequences of inquiry organized? And are there recognizable patterns to the interactional practices?

## Method

### Participants

Audio-recordings of three MBSR/MBCT classes taught by different teachers who consistently score proficient/advanced on the MBI:TAC (Crane et al. 2012) were analyzed. All follow the usual MBSR/MBCT format of eight 2–2.5 h weekly group sessions, with a daylong session of guided mindfulness practice during week 6 of the course. See Table 1.

**Table 1:** Demographic characteristics of intervention, teachers, and course participants

Group	Numbers of participants	Gender			Population	Program
		Teacher	Participants			
			M	F		
A	23	F	6	17	General public	MBSR
B	12	F	4	8	Trainee MBCT/MBSR teachers	MBSR
C	6	F	2	4	People with cancer	MBCT

### Procedure

Prior to commencement of the research, the study was reviewed and approved by the university’s research ethics and governance committee. The three teachers were collaborators and co-applicants. Consent was required from all course participants prior to commencement of recording and ceased if any participant decided to withdraw from the research. Recording of the entire 8-week course took place where possible. Recordings were stored on a password-protected hard drive and kept in a locked safe.

### Data Analysis

We aimed to adopt a stance of “unmotivated looking” as described by Psathas (1990) which is open to discovering interactional practices without being led by pre-formulated conceptualizations of what MB teaching should look like. To cultivate the capacity to simply listen with minimum intrusion of the conceptual mind and pre-formulated ideas about the teaching, the researchers sat in mindfulness meditation before reviewing each recorded exemplar. A key discipline during the analysis was to notice when we had moved beyond observation and into suggesting a motivation or intention for an interactional practice.

We started with 60 h of recorded material and then narrowed the focus to sections of teacher-led inquiry conducted after the first guided meditation practice in sessions 1–7 (approximately 7 h of material). The three researchers repeatedly listened to these inquiry sequences alongside reviewing first draft non-annotated transcripts, with the aim of identifying recurring conversational patterns. They then came together to collaboratively review recordings and transcripts. Sequences of interaction that offered examples of repeatedly observed interactional practices were selected and transcribed using a simplified CA transcription notation (Hutchby and Wooffitt 2009). The notation aims to capture all of the qualities present in actual speech, such as length of silences, overlapping speech acts, and qualitative features such as rising pitch, volume, added stress, and noises and utterances by speakers other than words (Table 2 details the transcribing conventions used). These extracts were then analyzed collaboratively by the researchers to enable detailed investigation of the interactional patterning of sequences.

**Table 2:** CA transcription symbols used (simplified from Hutchby and Wooffitt 2009)

[	Starting point of overlapping speech
=word	No break or gap between words or turns
(3.0)	Silence measured in seconds
(.)	Pause of less than 0.2 s
word	Emphasis
°word°	Especially quiet
wo:rd	Prolongation of sound
WORD	Words in capitals mark a section of speech noticeable louder than that surrounding it
wo-	Cut off
.hhh	Inhalation
↑↓	Shifts into especially high or low pitch

In this study, we gathered examples of MB teacher–participant interaction which were available to us and built up a corpus of inquiry sequences. We have not used random sampling or statistical sampling of populations to ensure the generalizability and representativeness of our findings. Peräkylä (2004) suggested that the “backbone” of CA work involves “qualitative case-by-case analysis” (p. 299). We analyzed a small number of cases, illustrating a specific form of MB teacher–participant interaction.

The reliability of the study is mainly ensured by examining naturally occurring interaction, rather than using researcher-prompted interactions (i.e., interviews) which then need to be generalized to everyday life. The data captures what actually happened in these MB courses.

To ensure the validity of the analytic claims, we did the following. Firstly, the first three authors made transcriptions and analytic observations independently and then collectively transcribed and analyzed the data extracts we selected. The three teachers reviewed the findings in light of their practitioner experience of the teaching process. Secondly, we adopted the standard CA practice of looking at how participants themselves display their understanding of what is happening in interaction, particularly the actions being performed in previous turns at talk. This is a crucial way of establishing validity because we could check our claims against the understandings displayed by participants.

## Results

Material from all three teachers was included evenly in the analytical process, but the extracts presented in the paper are teachers B and C. Extracts were chosen on the basis that they represent repeatedly seen conversational patterns, were succinct, and could make sense to a reader outside the overall context of the conversation that had preceded the extract.

There are a wide range of interactive patterns involved in inquiry. Here, we focus on three overarching practices which we observed repeatedly across the whole data set: turn-taking talk that involves questions and reformulations; talk that develops participants’ competence in a specific way of talking about experience; and talk that reinforces intersubjective connection and affiliation. The observed features of these three interactional practices are presented below and are illustrated through reference to numbered lines on transcript examples. The interactional practices are interrelated so overlap is inevitable. In each of the three sections below, therefore, priority is given to highlighting the key features of the practice under consideration, but aspects of the other two are also mentioned (T = teacher; A, B, or C = group; P = participant numbered in the order of first to speak).

1. Turn-Taking Talk that Involves Questioning and Reformulations

A turn-taking feature of generic pedagogical discourse contexts is a three-turn sequence in which the teacher asks a question (first turn), followed by the participant(s) answer (second turn), and then the turn routinely goes back to the teacher who gives a response (third turn) (Lee 2007). We found this characteristic three-turn sequence to be consistently employed by the MB teacher during inquiry sequences. Participants usually self-select to respond and shape the teacher’s third-turn response by choosing the timing and content of their second-turn contributions. A range of methods of teacher first- and third-turn responses were observed with a common theme of reformulating participants’ talk to satisfy the institutional aims of the MB course.

Extract 1 (session 2)
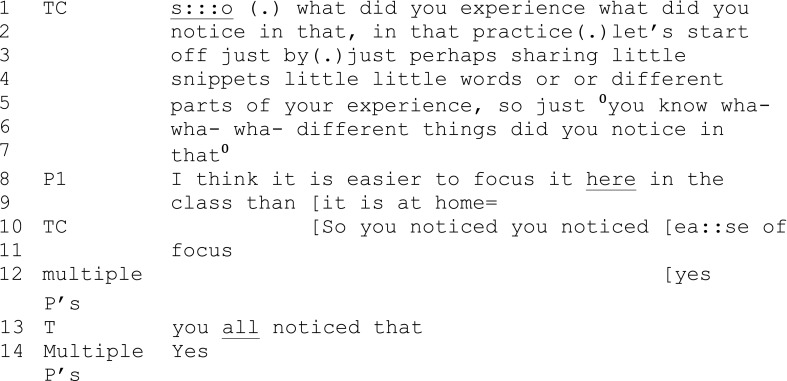


In extract 1, the teacher offers a complex first-turn part which contains a number of rephrased questions and instructions for the participants (1–7). In questioning the participants, the teacher also does some instructing work: establishing what is expected and required of participants in their next turn. The teacher alternates between a permissive, tentative openness which allows whatever was “experienced” or “noticed” to be shared (“perhaps sharing,” “different things”) and a prescriptive instruction about what is required of them (“let’s start off just by … just … little snippets”). The teacher limits the type of preferred range of possible response options and specifically directs the participants to talk about what they noticed in their experience as opposed to, for example, asking for feedback on the exercise or an evaluative question like “how well did you manage that?”

When a participant offers a second turn that is an evaluative, comparative response (8), the teacher is quick to offer a third turn (10). She talks over the participant and rather than responding directly to the evaluative aspect of the talk (i.e., class vs. home) she subtly reformulates what the participant has said by redescribing the participants’ recent experience: the comparative “easier,” which might have been developed into a story, becomes an emphasized and stand-alone “ease of focus.”

This reformulation does at least two further things. First, it is affiliative, in the sense that the teacher echoes the participants’ own words (easier) with a slight, but significant, modification (ease) that is actually a repair that redirects the participant’s offered focus, while at the same time doing description and acknowledgment rather than challenge or evaluation. Second, it successfully generalizes to, and includes, the group (note the multiple-participant “yes” in lines 12 and 14). The teacher then broadens the reformulation to the whole by giving emphasis to “all” (13). This interactional pattern of widening the learning outcomes from one individual’s experience to the group was frequently seen.

Extract 2 (later in same session from which extract 1 was taken)
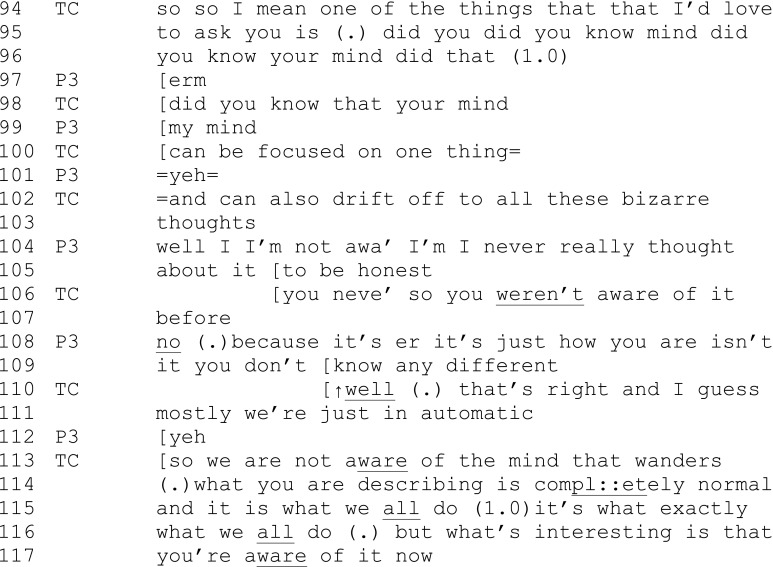


Extract 2 also shows how third-turn responses are used to widen the learning to the whole group following a series of turn-taking exchanges with one participant. In the lead up to this exchange, the teacher and participant 3 have together recreated her memory of her experience of her mind repeatedly being carried away with “bizarre thoughts.” In extract 2, the teacher and participant collaboratively construct the idea that the participant was not aware of her mind drifting to “bizarre thoughts” (see “thought” (104) repaired to “aware” (106, 113, 117)).

The teacher opens the exchange with a first-turn question, prefaced by a demonstration of keen curiosity (“love to ask you”) in the learning theme that she is directing the conversation toward (knowing that the mind wanders). In a series of closely overlapping turns (97–104), the participant responds to the teacher’s questions with recognition that she had “never really thought about it.” The teacher then introduces this as an example of the key theme of this stage of the program: “automatic” (111). She then extends this reformulation to others (“we are not aware” (113)), normalizes the experience (114), and communicates (through demonstrating her own curiosity) that this new awareness is a significant piece of learning (116–7). Note how the participant moves from “I” (104) to the less personal “you” (108), demonstrating her shifting sense that this is not a personal phenomenon. Thus, there is a co-constructed interactional build up to the learning point of universality, which the teacher directionally steers and participants collaborate in.

In summary, the turn-taking process is a co-construction in which the teacher opens the dialogue, a series of turns take place during which participants’ memories of their experience are collaboratively re-constructed, and then the teacher gathers, expands, and reformulates the learning for the benefit of the whole group. This gathering process sometimes takes place after a turn-taking sequence with one participant and at other times after a series of turns with several participants. The teacher determines the end point for the series of turns of questions and answers.

When the teacher offers third-turn reformulations of participant experience for the whole group, they sometimes keep their turn for an extended time. Thus, didactic teaching follows on and is linked to themes that participants have already introduced. Learning themes are only introduced as examples of them emerge in participants’ accounts of their specific experience.

2 The Development of Skills in a Particular Way of Describing Experience

Participants’ talk is shaped by the teacher toward the conversational norms of a MB class. This can be seen in action within the talk in a number of ways.

Extract 3 (which occurs in session 2 between extracts 1 and 2)
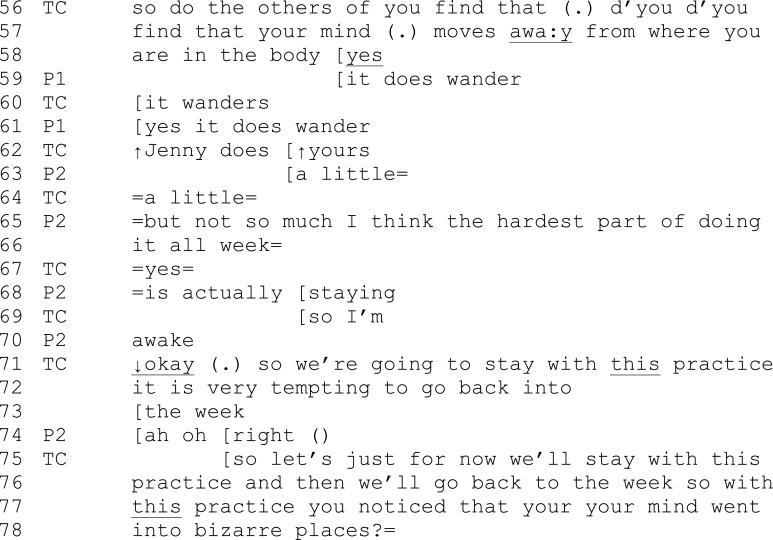


Extract 3 shows how the teacher directionally leads the focus of the conversation back to the sorts of areas of focus for a MB class—in this particular instance to a focus on specific experience in a specific practice. Following an exchange with a participant who is experiencing difficulty with mind wandering in the practice, the teacher widens the exploration to the whole group (56–8). The question builds toward the theme of universality through appeal to others’ experiences. However, participant 2 takes the interaction away from a specific exploration of experience within recent practice (65–70). The teacher interrupts and overlaps with the participant with an emphatic “Okay” (71) before bringing the focus back (71–6) with the highly directive “so we are going to stay with this practice” with its emphatic “this.” The teacher continues with a softer affiliative tone and an invitation to “just for now stay” (75). The teacher redirects the focus of discussion back to the recent practice.

In summary, a range of interactional practices through which the teacher aims to train participants’ competence in describing their experience in ways consistent with mindfulness practice were seen in the data:

First, participants are learning to anchor their learning in specific direct experience rather than in generalized ideas about experience, and when participants become less specific, they are firmly redirected.

Second, participants generally only speak about their own immediate experience, so they predominantly use the pronoun “I,” are redirected when they generalize beyond their own experience, and are not generally given space to elaborate about their experience. The teacher draws out themes that are likely to be universal to everyone, so during the third-turn reformulations, they make a pronoun shift to “us” and “we” (see 113–7, extract 2) and speak about “the” mind rather than using the possessive “your” mind (see 113, extract 3). Generally, the teacher is the member of the group who is given space to generalize experience in these ways.

Third, participant talk that is a detailed, rich, non-analytic account of a specific and recent example of immediate experience that relates to key learning themes produces greater displays of interest from the teacher and to longer time sequences of turn-taking with the teacher. In extract 2, the teacher uses each turn with participant 3 as an invitation to display key learning themes to the whole group. By contrast, when participants are not offering contributions that fit, teachers either explicitly redirect the interaction or offer short emphatic minimal response tokens which project for an end to the turn-taking (see the “okay” at line 71 in extract 3).

Fourth, teachers appear to be training participants to display interest and curiosity in ordinary everyday experience and in mind patterns that might have previously been off radar (see 94–6 in extract 2 where participant 3 is asked if she has noticed mind wandering and expresses that she had never thought of it before (104–5)). The teacher is actively directing participants toward recognition that in this context the apparently ordinary becomes an important topic, and socially normative responses are descriptive reports about noticing recent direct experience.

Fifth, participants are being trained to speak in the language of “noticing” and “being aware of” experience (e.g., see 2, 6, 10, 14 in extract 1) as they retrospectively co-construct their experience using language. In other recordings, teachers would specifically reward participants’ noticing skills with the expression “good noticing”, and noticing of a “small” experience is rewarded with an emphasized positive assessment.

3 Talk that Constructs Intersubjective Connection and Affiliation

As has been noted in Sections 1 and 2 above the MB teachers’ talk seems designed to produce a sense of affiliation and connection both between teacher and participant, and across the whole group (including the teacher), through a repeated practice of constructing a connection with the universality of human experience (e.g., extract 3, lines 113–117).

Extract 4 (session 4)
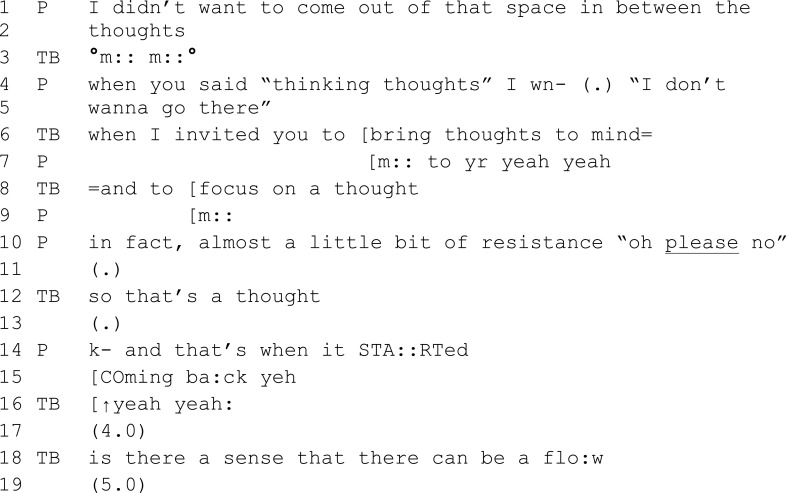


Extract 4 shows teacher–participant affiliation being created through interaction. The teacher validates the participants’ experience and communicates affiliation through the soft, long “mmm” (3). There are regular, and often long, pauses (11, 13, 17, 19) which may demonstrate willingness of both the teacher and the participant to stay with her experience and that this is an okay place to be together. Notice also how teacher and participant match each other’s pace and tone (see the rising intonation of the teacher in 16 which follows the participant’s emphasis on “coming”).

The teachers generally used many highly positive minimal response tokens (Jefferson 1984) (words, such as “right,” “sure,” “yeh,” “mm”) often overlapping with participants’ talk. Here, in extract 4, the teacher’s “mm’s” overlap with the participant’s words and in this instance communicate affiliation and actively signal to the participant to continue because what she is saying is of interest and value. As expressed earlier, these minimal responses can also be used to close down and change topic (see the “okay” in 71, extract 3).

Extract 5 (session 2) (picking up a short while after extract 1)
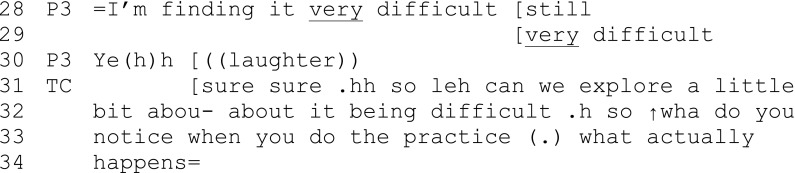


In extract 5, the teacher offers an echo of tone and content of the phrase “very difficult,” performing a sense of alliance and affiliation with participant 3 as she shares her experience of challenge and communicates difficulty (though interpolated laughter). The participant is able to come in with a completely contrasting experience (28), implying that (although this is only session 2) an encouraging, invitational atmosphere has been established and is continually being reinforced through conversation. In other session recordings, the teachers explicitly encourage participants to share experiences that might “be the same as or different to” what has just been shared.

Although the sequences of transcript tend to be between the teacher and one other participant, it is important to remember that every conversation happens in the presence of the whole group. The teachers frequently use strategies during a turn-taking sequence with one participant to ensure wider participation, to encourage affiliation between everyone present, and to encourage recognition that the theme being explored is relevant to everyone.

## Discussion

A key aim of this research was to explicate the context-specific structural organization of talk in MB inquiry and to pilot the potential of the CA method for this context. The analysis aimed to make visible some of the taken-for-granted, implicit, or unrecognized practices that take place during inquiry. It makes a unique contribution by using CA to reveal a particular perspective on the complex and multi-faceted moment-by-moment practice of inquiry in MBSR/MBCT teaching. This offers insights into the pedagogic relationship between the participants’ development of understanding and the interactional practices used. Further work is needed to investigate how these understandings relate to participant outcomes.

It is clear that the contingencies of the social organization of the participatory learning process influence the shape and character of each moment. We suggest that these implicit but important social processes have a significant influence on participant learning. It is therefore important to recognize and account for them in teaching and in the training of teachers. CA offers a methodology for unpacking these taken-for-granted processes and revealing the practical enactment of the teachers’ pedagogical work.

One specific strand of CA, known as institutional CA, focuses on communicative practices that are specific to particular workplace settings. Drew and Heritage (1993) articulated three features of talk in workplace settings: it is goal orientated in relevant ways; it involves particular and special constraints on allowable contributions; and it is associated with particular frameworks and procedures. The findings demonstrate that MB inquiry has context-specific aspects within each of these features: inquiry has a particular direction and purpose which is aligned with the aims of the course and which are firmly maintained by the teacher; linked to this, the teacher shapes the norms of what content is included and excluded from the process; and there are particular interactional frameworks, methods, and structures which enable dialogic exchange to take place.

Simultaneously, however, there is an ongoing process of improvisation taking place. In the process of repeated participation, the teacher continually responds to the process and modifies their methods to achieve the task in hand. In contrast with some other teacher-led whole class dialogue contexts, the MB teacher is not seeking a specific right response, is not the primary knower of information, genuinely does not know the participants’ experience, and cannot therefore know in advance the exact learning themes that will emerge in the moment. The co-construction is therefore highly specific to the conditions of the moment. The teacher’s skill in being able to dance with the emergence of each moment while steering the learning process is of paramount importance. This underlines both the importance of planning and preparation before a class is taught and of letting go into the actuality of each moment (rather than being constrained by ideas of how it could or should be), so that it is possible to be responsive to the moment. The term “disciplined improvisation” coined by Sawyer (2004) aptly describes the creative tension inherent within the process.

The balance between creating an open welcoming atmosphere and steering the teaching process in a strongly directional way is delicate. Participatory dialogic teaching requiring turn-taking needs to be carefully managed; learning tends to be more effective when it is co-constructed, and there is active involvement from participants to shape the direction of the discourse (Radford et al. 2011). The teacher is making continuous micro-judgements about how much floor space to give to a particular participant contribution. The overriding pedagogic goal is to lead an emergent process of dialogue that has enabled each contributor to deepen their understanding and that leads to a shared understanding that everyone has been party to (Skidmore and Murakami 2012).

The pedagogical literature on MB approaches emphasizes the implicit qualities that MB teachers need to embody. The inquiry process rests not only on these deeply embodied understandings of mindfulness practice within the teacher, but it also relies on the teacher’s capacity to enact the multiple skills required to manage participatory classroom dialogue. No matter how profound any piece of MB teacher communication is, it is only as effective as the response it generates and therefore needs to be understood in the conversational context it was made in. Through CA, these interactional skills can become tangible, visible, and explicit. Analysis of these explicit competencies offers a view on how the implicit qualities of mindfulness emerge as learning themes within the teaching process. For example, mindfulness-based teachers seem to have a particular way of hearing experience. That is to say that they are able to scan the detail of expressed experience and hone the focus of inquiry to a particular aspect that is relevant to the learning process. The particular skills and knowledge required to enact this competence are not well articulated in the literature, but in our view, much of it rests on a moment-by-moment connection to the teacher’s personal mindfulness practice, which they embody during teaching.

Psathas (1990) proposed “the interactional phenomena that are discovered…will enable us to state with greater certainty, what interactional competencies are requisite… And if members are lacking particular identifiable and describable interactional skills, we should be able to develop methods for teaching, demonstrating or training” (p. 21). Each moment of inquiry is unique; however, CA enables us to see that repeating patterns of interaction in inquiry can be made visible. Trainee MB teachers commonly share that leading the inquiry process in MB teaching is the most challenging and daunting aspect of the overall learning process. They could become more attuned to the practices at play within inquiry through the integration of perspectives from CA (using recorded examples of teaching) into training.

### Limitations and Future Directions

Doing a conversation analysis reveals a lot of delicate subtle interactional work on the part of teachers and participants. The limitations of the scope of this study mean that only a small amount of this highly sophisticated and nuanced work could be selected for noticing and analysis here. In particular, the interactions of only three teachers were analyzed. Furthermore, we have only examined what is hearable within the teaching—there are clearly other (seeable and feelable) dimensions and processes that are taking place that are not included here. One dimension which could be included in a future study using CA methods is the visual aspects of communication. In the context of MB teaching, this could be a particularly useful way to examine how mindfulness is communicated through the teacher’s embodiment of process.

In this study, we used CA in a relatively wide angle way to reveal some general characteristics of MB inquiry. This limited the level of detailed analysis of each practice. We suggest that CA techniques offer the potential to move into narrow angle-detailed analysis of themes within each area. For example, the teacher has a range of options with the third-turn response. Detailed analysis of these and their consequences would be informative. For this study, we chose extracts which demonstrated typical features. It would be useful to investigate moments when the unexpected happens and when tensions arise in the learning process. The CA transcription system offers opportunity to study how the emotional climate is co-created in the classroom. We saw some shifts in the interactional practices employed by teachers and participants over the 8 weeks as the competencies of participants grew, but the scope of this study did not enable analysis of this. An exploration of how this happens would enable greater understanding of the tasks of the teacher at different stages in the program. Our study revealed that the MB teaching process is highly directional and teacher-led. There is an interesting tension between directional leadership and participatory co-construction that is at play in the teaching process. How this is navigated by the teacher could be a specific focus. A significant portion of the learning process in MB courses takes place in dyads and small groups. The CA approach could be extended to investigate discourse between participants without the direct intervention of the teacher.

Different research methods require the researcher to stand in different relationship to the object of study. The MB teaching process has been evaluated by third-person explorations, which aim to objectively describe and assess the pedagogical process (Crane et al. 2013). In this study, we have employed a second-person examination of intersubjective, interactional practices. There is further potential to use first-person research methods by examining teachers’ reflections on internal processes as they teach (e.g., via diaries/interviews). A research process which examined a piece of teaching by triangulating findings from these three perspectives has the potential to be particularly rich.

## Conclusions

While it is essential to draw on theory and on the wisdom of expert teachers, it is also essential to build an empirically based body of knowledge about the MB teaching process and a collection of valid empirical methods for conducting research in this area. In this first study of MB teaching to examine naturally occurring material rather than retrospective accounts of participant experience or expert views of the teaching process, we have used CA to investigate what teachers and participants actually do with their talk in MBSR/MBCT. This revealed how turn-taking happens and how the teacher reformulates participant contributions; particular participant competencies that are being trained through dialogue; and the atmosphere of affiliation that is created through the process of interaction in the group. Use of CA reveals the complexity of the interactional work that MB teachers are engaged in when leading participatory dialogue.

There have been some understandable concerns expressed in the field that the positivist outcome-focused research agenda while promising on one level could lead to teachers delivering the course who are also primarily focused on outcome, rather than being deeply immersed in the practice of mindfulness on which the whole pedagogy is based. There is a significant imbalance between the large and rapidly expanding outcome evidence base for MB approaches and the surprisingly small empirical literature on the pedagogy by which these effects are arguably created. In our view, the development of a carefully thought through research agenda and empirical literature specifically focussed on investigating the process, and intrinsic qualities of MB teaching would greatly support further thinking on teaching integrity and on teacher training and development, which is much needed in the context of growing demand for training. It is a challenge that we hope the field will embrace so that there is a growing literature that represents the disciplined improvisation that is the work of the MB teacher.
